# Developing a framework for estimating comorbidity burden of inpatient cancer patients based on a case study in China

**DOI:** 10.1186/s41256-025-00411-3

**Published:** 2025-03-03

**Authors:** Jiamin Wang, Wenjing Zhang, Kexin Sun, Mingzhu Su, Yuqing Zhang, Jun Su, Xiaojie Sun

**Affiliations:** 1 Centre for Health Management and Policy Research of Shandong University (Shandong Provincial Key New Think Tank), Jinan, 250012 China; 2 NHC Key Lab of Health Economics and Policy Research, Shandong University, Jinan, 250012 China; 3 Infection Management Department, Longquanyi District Traditional Chinese Medicine Hospital, Chengdu, 610100 China; 4 Office for Cancer Registry, National Cancer Center/National Clinical Research Center for Cancer/Cancer Hospital, Chinese Academy of Medical Sciences and Peking Union Medical College, Beijing, 100021 China; 5 Department of Social Medicine and Health Management, School of Public Health, Cheeloo College of Medicine, Shandong University, Shandong, China

**Keywords:** Comorbidity, Inpatient, Cancer, Case study

## Abstract

Inpatient cancer patients often carry the dual burden of the cancer itself and comorbidities, which were recognized as one of the most urgent global public health issues to be addressed. Based on a case study conducted in a tertiary hospital in Shandong Province, this study developed a framework for the extraction of hospital information system data, identification of basic comorbidity characteristics, estimation of the comorbidity burden, and examination of the associations between comorbidity patterns and outcome measures. In the case study, demographic data, diagnostic data, medication data and cost data were extracted from the hospital information system under a stringent inclusion and exclusion process, and the diagnostic data were coded by trained coders with the 10th revision of the International Classification of Diseases (ICD-10). Comorbidities in this study was assessed using the NCI Comorbidity Index, which identifies multiple comorbidities. Rates, numbers, types and severity of comorbidity for inpatient cancer patients together form the characterization of comorbidities. All prevalent conditions in this cohort were included in the cluster analysis. Patient characteristics of each comorbidity cluster were described. Different comorbidity patterns of inpatient cancer patients were identified, and the associations between comorbidity patterns and outcome measures were examined. This framework can be adopted to guide the patient care, hospital administration and medical resource allocation, and has the potential to be applied in various healthcare settings at local, regional, national, and international levels to foster a healthcare environment that is more responsive to the complexities of cancer and its associated conditions. The application of this framework needs to be optimized to overcome a few limitations in data acquisition, data integration, treatment priorities that vary by stage, and ethics and privacy issues.

**Supplementary Information:**

The online version contains supplementary material available at 10.1186/s41256-025-00411-3.

## Introduction

Cancer is a major global public health issue. Nearly 20 million people were diagnosed with cancer in the year 2022 alongside 9.7 million death [1, 2]. Global cancer statistics predict that the number of new cases of cancer will reach 35 million by 2050. Inpatient cancer patients often carry the dual burden of the cancer itself and other coexisting chronic conditions (comorbidity), which is also recognized as one of the most urgent global public health issues to be addressed [3, 4]. The relationship between cancer and comorbidity is multidimensional and cancer shares common risk factors with most chronic diseases, like advanced age or unhealthy lifestyles. For instance, consuming high levels of alcohol and carrying excess body weight increase one's susceptibility to cancer and chronic ailments like diabetes or cardiovascular disease; similarly, both chronic obstructive pulmonary disease (COPD) and lung cancer are known to be heavily influenced by tobacco use [5, 6].

Comorbidity in cancer has been established as one of the key predictors of poor prognosis [7]. It may influence cancer survival by complicating treatment options, increasing cost of care, decreasing quality of life, camouflaging the cancer symptoms and causing diagnosis delay [8, 9]. Effective and tailored inpatient cancer care interventions are formulated based on understanding the patterns of diseases that coexist with cancer and investigating its impact on cancer treatment and prognosis. The comorbidity pattern varies across the stages of the cancer journey, from diagnosis and treatment to survival. Therefore, a general framework is needed to estimate the comorbidity burden unique to each cancer stage.

Numerous methods have been utilized to analyze the comorbidity issue for cancer patients in different cancer stages, such as prevalence statistics [10, 11], network analysis [4, 12, 13], association rule mining [14, 15], and latent class analysis [16]. However, due to the lack of a general framework, the conclusions are heterogeneous. Existing published guidelines on comorbidities mainly focus on the treatment stage. Guidelines published by the American Geriatrics Society in 2012 [17] and National Institute for Health and Clinical Excellence in 2016 [18] all proposed that interpretation should be individualized for patients with comorbidities, and clinical decisions should be made by weighing the benefits, risks, burdens, and prognosis. To systematically explore the hidden information of comorbidity burden for inpatient cancer patients, a framework was developed in this research based on a case study conducted in X tertiary hospital in Shandong Province.

## Development of the framework

**Fig. 1:**
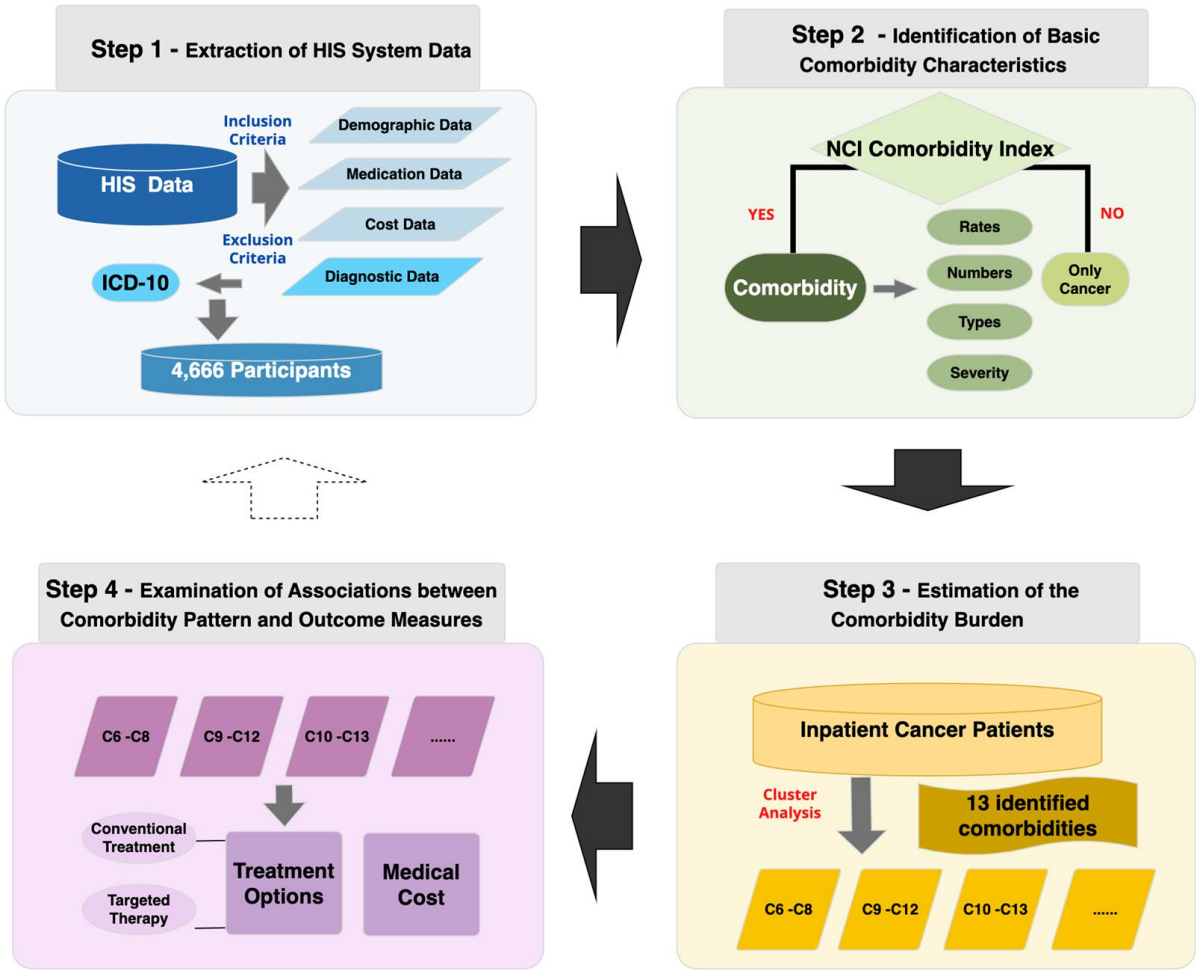
Flow chart of the framework for Estimating Comorbidity Burden of Inpatient Cancer Patients

Figure 1 presents a framework for estimating the comorbidity burden of inpatient cancer patient, which consists of four steps. The first step is the extraction of HIS system data like demographic data, diagnostic data, medication data and cost data. The inclusion and the exclusion process were stringently restricted. The diagnostic data were coded by trained coders with the 10th revision of the International Classification of Diseases (ICD-10). A total of 4,666 patients were finally included in the analysis. The second step is the identification of basic comorbidity characteristics. Comorbidities in this study were assessed using the NCI Comorbidity Index. Rates, numbers, types and severity of comorbidity for inpatient cancer patients together form the characterization of comorbidities. The third step is the estimation of the comorbidity burden. Inpatient cancer patients were classified based on comorbidities according to the agglomerative hierarchical cluster analysis. All prevalent conditions in this cohort were included in the cluster analysis. The final step is the examination of the associations between comorbidity patterns and outcome measures which include treatment options and medical cost. Treatment options were divided into conventional treatment and targeted therapy. More detailed information of the framework development was shown in the following part.

### Study site

Shandong province is an important coastal province with over 100 million people in East China. The trend of incident cases and death of cancer in Shandong Province is consistent with that of the. whole country, among which lung cancer, gastric cancer, colorectal cancer, esophageal cancer and liver cancer are the most common types of cancer. The case hospital founded in 1916 was one of the largest municipal hospitals in Shandong province and represented the regional highest level of oncology care.

### Step 1: extraction of HIS system data

**Fig. 2:**
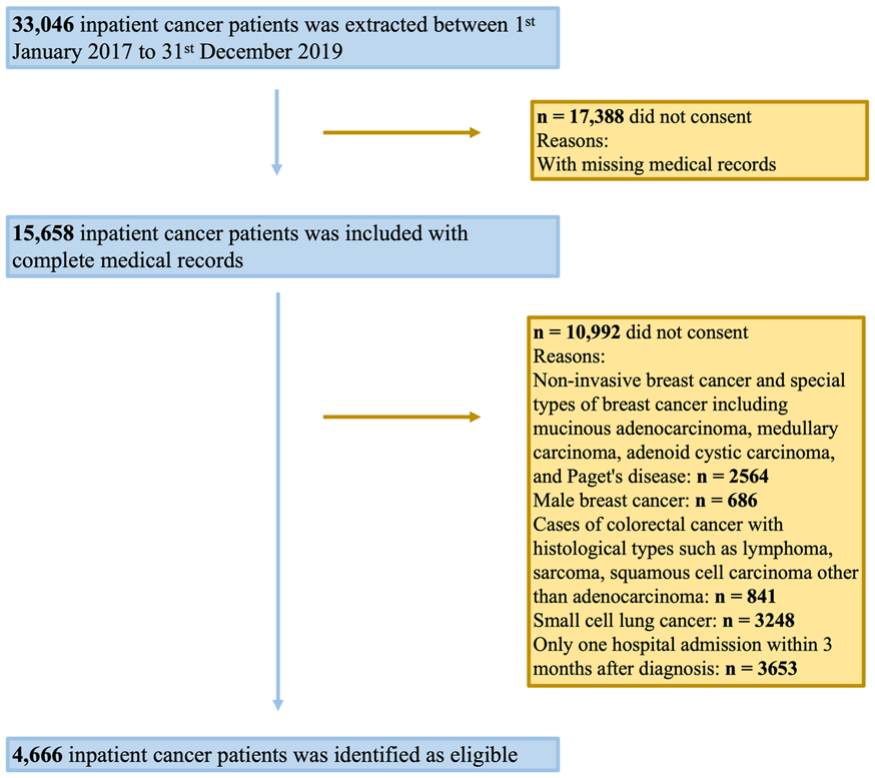
Flow chart of the included participants from the case hospital

The entire participant inclusion flowchart is illustrated in Fig. 2. By extracting the information from the hospital information system (HIS) of the case hospital, the data of all potential research objects were obtained. The inclusion criteria included: lung, colon, rectal, breast and gastric cancer cases confirmed by pathological examination; and the time of diagnosis was from January 2017 to October 2019. Cancer patients typically undergo follow-up examinations every 3–6 months. To maintain the integrity of data regarding patients’ treatment experiences, we excluded those who had only one hospital admission within a 3 months period. Furthermore, to account for the confounding effects of diverse pathological characteristics, we also excluded several specific patient groups due to their unique treatment requirements and relatively low incidence rates. These groups include: non-invasive breast cancer and special types of breast cancer including mucinous adenocarcinoma, medullary carcinoma, adenoid cystic carcinoma, and Paget's disease; male breast cancer; cases of colorectal cancer with histological types such as lymphoma, sarcoma, squamous cell carcinoma other than adenocarcinoma; and the small cell lung cancer [19]. According to the cancer type, patients in the departments of oncology, thoracic surgery, breast surgery, general surgery, and colorectal oncology in the case hospital were selected. The data contained more than 300,000 records, including demographic data (such as age and sex), diagnostic data, medication data and cost data. The diagnostic data consisted of one primary diagnosis and up to 18 secondary diagnoses, which were coded by trained coders with the ICD-10. A total of 4,666 patients were finally included in the analysis.

The characteristics of 4,666 cancer patients are presented in Table 1. Among all cancer patients, half of them were male (50.32%) and the average age was 62.42 (SD = 11.62) years. The majority of patients were insured (94.68%) and got married (87.74%). More than one third of patients were diagnosed with lung cancer (37.03%), followed by gastric cancer (21.20%), breast cancer (17.08%), rectal cancer (12.47%) and colon cancer (12.22%). Supplementary Table S1 presents the detailed information by cancer type. Breast cancer patients with the youngest average age (54.40) of all participants, while rectal cancer patients with the oldest (65.34). More gastric cancer (61.78%) and rectal Cancer patients (52.41%) were in the III or IV stage. Except for breast cancer, all cancer types were male dominated.

**Table 1:** Baseline characteristics of Chinese inpatient cancer patients included in this study

	Total (N = 4666)	Comorbidity		
		With (N = 3554)	Without (N = 1112)	*P* value
Age, years, mean (SD)	62.42(11.62)	64.53(10.63)	55.67(12.07)	< 0.001***
Cost, RMB, mean (SD)	73,551.74(65,287.61)	76,722.81(68,358.97)	63,416.87(53,090.68)	< 0.001***
Sex, n (%)				< 0.001***
Female	2318 (49.68)	1609 (45.27)	709 (63.76)	
Male	2348 (50.32)	1945 (54.73)	403 (36.24)	
Insured, n (%)				< 0.001***
Yes	4418 (94.68)	3393 (95.47)	1025 (92.18)	
No	248 (5.32)	161 (4.53)	87 (7.82)	
Married, n (%)				0.363
Yes	4094 (87.74)	3127 (87.99)	967 (86.96)	
No	572 (12.26)	427 (12.01)	145(13.04)	
Residency, n (%)				< 0.001***
Urban	3031 (64.96)	2408 (67.75)	623 (56.03)	
Rural	1635 (35.04)	1146 (32.25)	489 (43.97)	
Cancer type, n (%)				< 0.001***
Lung cancer	1728 (37.03)	1446 (40.69)	282 (25.34)	
Colon cancer	570 (12.22)	480 (13.51)	90 (8.09)	
Rectal cancer	582 (12.47)	457 (12.86)	125 (11.24)	
Breast cancer	797 (17.08)	385 (10.83)	412 (37.05)	
Gastric cancer	989 (21.20)	786 (22.12)	203 (18.26)	
Cancer stage, n (%)				< 0.001***
0	124 (2.66)	85 (2.39)	39 (3.51)	
I	1321 (28.31)	951 (26.76)	370 (33.27)	
II	997 (21.37)	668 (18.80)	329 (29.59)	
III	1358 (29.10)	1011 (28.45)	347 (31.21)	
IV	866 (18.56)	839 (23.61)	27 (2.43)	

^***^ *P* value < 0.001; ** *P* value < 0.01; * *P* value < 0.05

Numbers presented are as n (%) unless otherwise specified

### Step 2: identification of basic comorbidity characteristics

In this study, comorbidities were assessed using the NCI Comorbidity Index, which identifies multiple comorbidities. Based on the Charlson Comorbidity Index (CCI) first developed in 1987 by Mary Charlson and colleagues [20], the cancer-specific NCI Comorbidity Index developed by Carrie Klabunde and colleagues [21] excluded solid tumors, leukemias, and lymphomas as comorbid conditions, given that the NCI Comorbidity Index was developed from a cohort of cancer patients. The NCI Comorbidity Index was created to address some limitations of the Charlson Comorbidity Index, especially when applied to cancer patients. The remaining 16 Charlson index conditions were included in the NCI Comorbidity Index, with further consolidation to 14 conditions (Table 2): moderate/severe liver disease, cerebrovascular disease (CVD), peripheral vascular disease (PVD), renal disease, paralysis (hemiplegia or paraplegia), myocardial infarction, peptic ulcer, dementia, AIDS, mild liver disease, congestive heart failure (CHF), COPD, diabetes with complications, and diabetes. Adjusted specifically for cancer-specific NCI Comorbidity Index, the CCI accounts for multiple comorbidities according to the presence of 14 comorbid conditions. Hypertension was also included as it had the highest prevalence rate in the study sample, aside from those included in the NCI Comorbidity Index. For the severity of Charlson’s comorbidity, it was classified into mild, moderate and severe categories based on the CCI weight. Each condition was assigned a weight from 1 to 6, according to the estimated 1 year mortality hazard ratio from a cox proportional-hazards model. These weights were summed to produce the Charlson comorbidity score [22].

**Table 2:** Frequency of comorbidities in included Chinese inpatient cancer patients

Classification of Diseases	Prevalence (%)					
	Total	Lung cancer	Colon cancer	Rectal cancer	Breast cancer	Gastric Cancer
***Cancer-specific NCI Comorbidity***						
Mild Liver Disease	1046	397(37.95)	172(16.44)	154(14.72)	110(10.52)	213(20.36)
Renal Disease	821	264(32.16)	158(19.24)	163(19.85)	19(2.31)	217(26.43)
Diabetes	742	300(40.43)	106(14.29)	105(14.15)	79(10.65)	152(20.49)
Myocardial Infarction	730	292 (40.00)	119 (16.30)	85 (11.64)	68 (9.32)	166 (22.74)
Peptic Ulcer	442	153 (34.62)	77 (17.42)	55(12.44)	29 (6.56)	128(28.96)
Peripheral Vascular Disease (PVD)	379	172 (45.38)	56 (14.78)	60(15.83)	30 (7.92)	61 (16.09)
Chronic Obstructive Pulmonary Disease (COPD)	358	199 (55.59)	30(8.38)	52 (14.53)	17 (4.75)	60 (16.76)
Cerebrovascular Disease (CVD)	235	110 (46.81)	36 (15.32)	24 (10.21)	13(5.53)	52(22.13)
Moderate/Several Liver Diseases	90	28 (31.11)	14 (15.56)	14 (15.56)	8 (8.89)	26(28.89)
Congestive Heart Failure (CHF)	77	33 (42.86)	9 (11.69)	11 (14.29)	0 (0)	24(31.17)
Diabetes with Complications	49	29 (59.18)	7 (14.29)	4 (8.16)	2 (4.08)	7 (14.29)
Connective Tissue Disease	39	22 (56.41)	4(10.26)	2 (5.13)	5 (12.82)	6 (15.38)
Dementia	5	0 (0)	0 (0)	2 (40)	1 (20)	2 (40)
Paralysis (Hemiplegia or Paraplegia)	2	0 (0)	2 (100)	0 (0)	0(0)	0 (0)
***Other common comorbidity***						
Hypertension	1495	601(40.20)	222(14.85)	196(13.11)	171(11.44)	305(20.40)

^***^ *P* value < 0.001; ** *P* value < 0.05; * *P* value < 0.1

Numbers presented are n (%) unless otherwise specified

Sociodemographic and cancer characteristics were compared according to the number of comorbidity and the severity of comorbidity. Continuous variables were summarized as mean (SD) and were examined using the Student t test. Categorical variables were presented as the proportion (%) and compared using the Pearson chi-square test. Of the 4,666 participants, there were more patients (76.17%) with comorbidities than those without. Compared with those without comorbidities (Table 1), patients with comorbidities were older (64.53 vs 55.67 years, *P* < 0.001), more male (*P* < 0.001), more urban lived (*P* < 0.001), more lung cancer diagnosed (*P* < 0.001) and more III-cancer-stage (*P* < 0.001). In terms of the number of comorbidities (Supplementary Figure S1), among 3554 cancer patients with comorbidities, 97.36% of patients had less than three types of comorbidities, of which 37.03% of patients had one comorbidity, 29.04% had two, and 17.14% had three. Compared with those with less than three types of comorbidities (Supplementary Table S2), patients with more than three types of comorbidities were older (*P* < 0.001) and in later cancer stage (*P* < 0.001). The most common comorbidities were hypertension (32.04%), mild liver disease (22.42%), renal diseases (17.60%), diabetes (15.90%) and myocardial infarct (15.65%). For the severity of comorbidity, more than half of patients were with the severe comorbidities (69.11%). Most patients with the severe comorbidities were in the IV cancer stage (81.57%), living in urban areas (67.49%), and diagnosed with lung cancer (56.69%).

### Step 3: estimation of the comorbidity burden

**Fig. 3:**
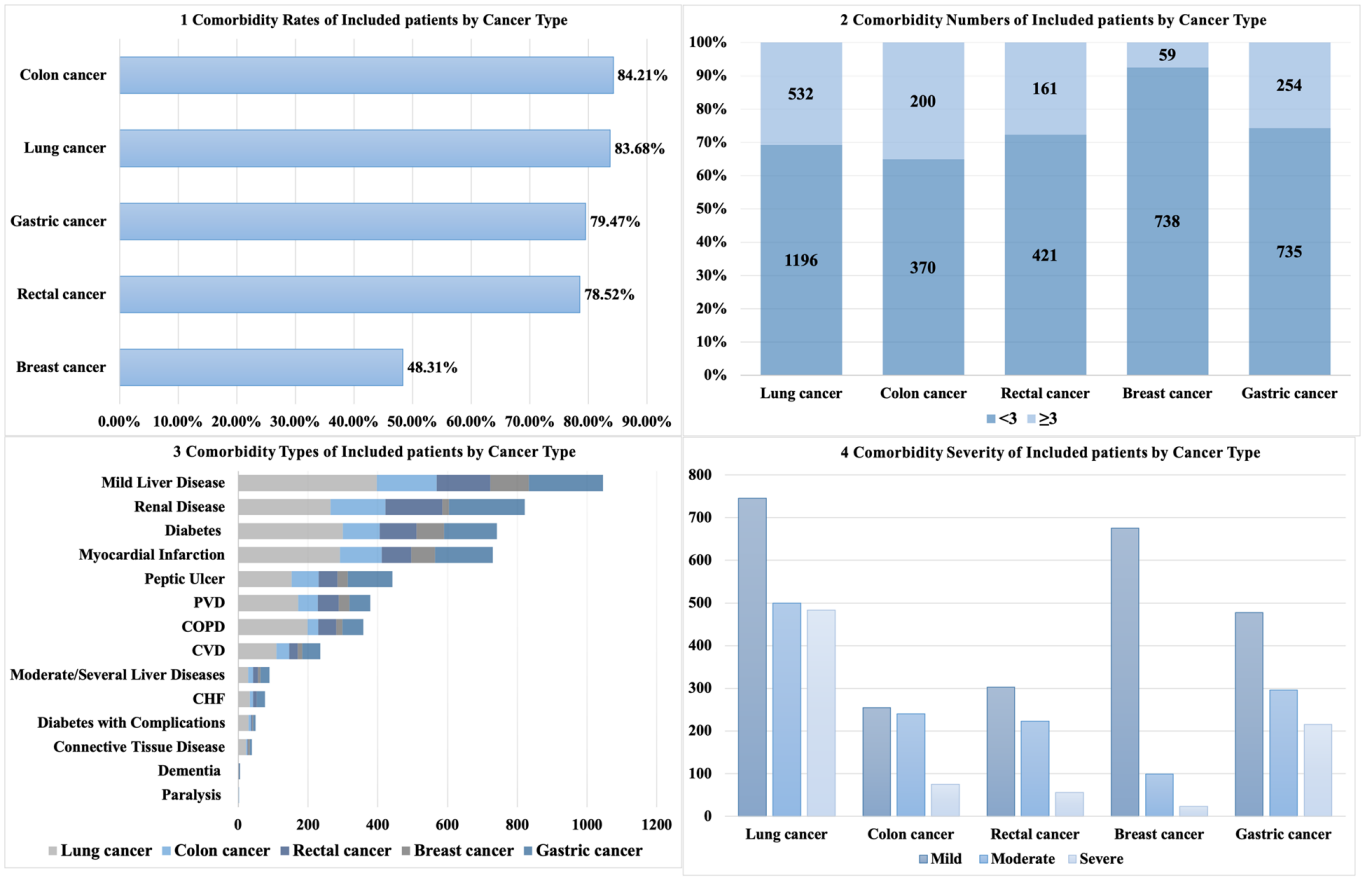
Comorbidity Burden of included Chinese inpatient cancer patients by Cancer Type (N = 3554)

Figure 3 demonstrated the rates, numbers, types and severity of comorbidity for inpatient cancer patients by cancer types. The highest comorbidity rate was observed in colon cancer patients (84.21%), followed by lung cancer (83.68%), gastric cancer (79.47%), rectal cancer (78.52%) and breast cancer (48.31%). Most cancer patients had fewer than three types of comorbidities across all cancer types, with the highest proportion observed in breast cancer patients (92.60%). Hypertension and mild liver disease were the most prevalent comorbidities across all five cancer types. Diabetes was the third most common comorbidity in lung (17.36%) and breast (9.91%) cancer patients, while renal disease ranked the third in colon (27.72%) and rectal (28.01%) cancer patients. A significant proportion of lung (56.89%), colon (55.26%), and gastric (51.69%) cancer patients had moderate to severe comorbidities.

**Fig. 4:**
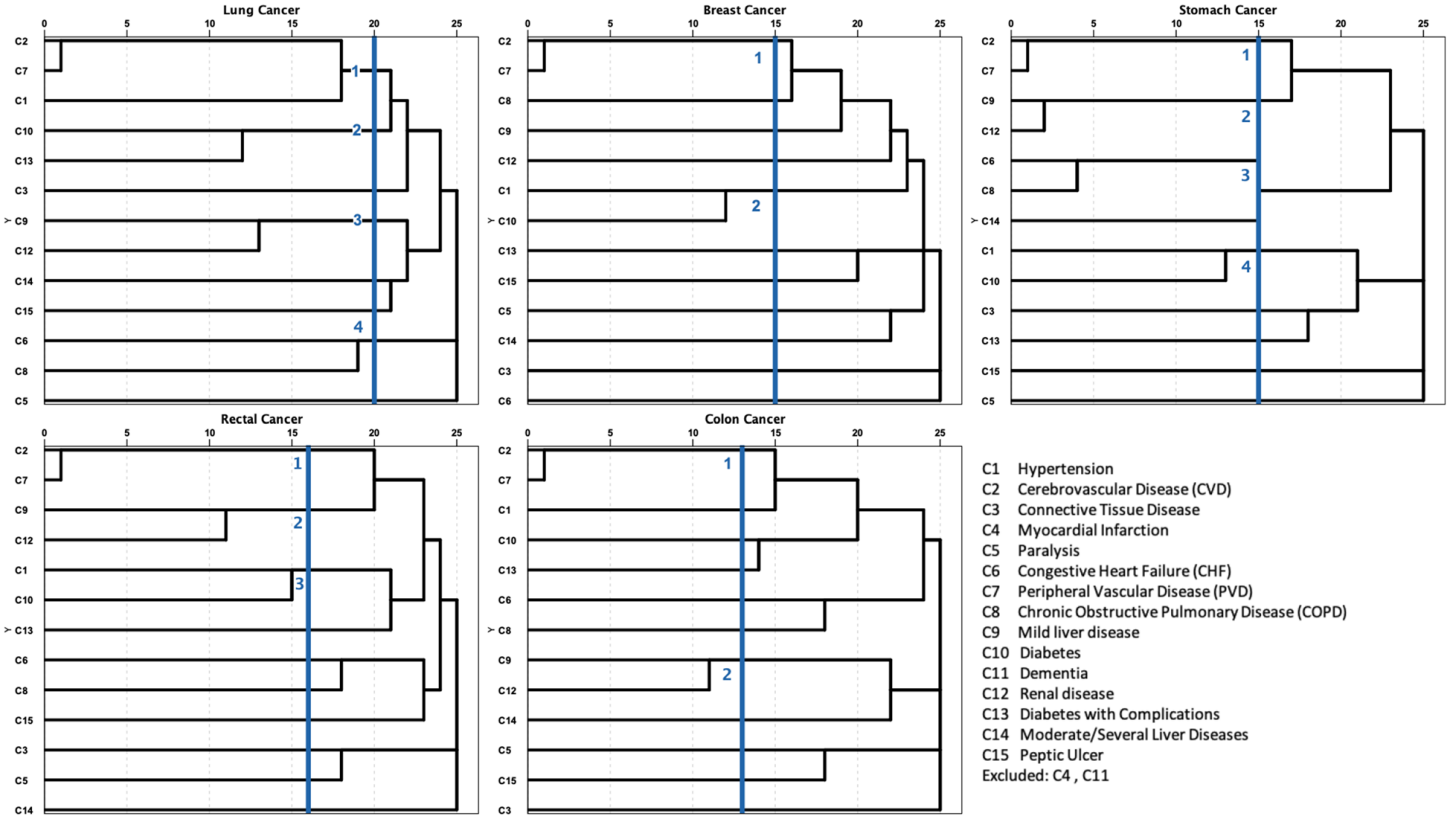
Comorbidity Pattern of included Chinese inpatient cancer patients (N = 3554)

Agglomerative hierarchical cluster analysis [23, 24] was used to classify individuals into groups based on comorbidities, and this was a commonly used bottom-up clustering method which clustered from individual patients to a final group containing all patients. Pearson correlation coefficient was used as a measure of distance between data points, and the data were standardized to convert the correlation into a distance measure, where data points with higher correlation were closer together and those with lower correlation were farther apart [25]. All prevalent conditions in this cohort were included in the cluster analysis. Patient characteristics of each comorbidity cluster were described. Among all 15 identified comorbidities, only 13 conditions were included in the comorbidity pattern analysis. Myocardial infarction (n = 0) and dementia (n = 2) were excluded due to the limited number of reported inpatient cancer patients. The diagram from cluster analysis of the remaining 13 conditions by five types of cancer is shown in Fig. 4. For lung cancer patients, four categories of comorbidities were identified: C6 CHF—C8 COPD (n = 14), C1 hypertension—C2 CVD—C7 PVD (n = 74), C9 mild liver disease—C12 renal disease (n = 56), and C10 diabetes—C13 diabetes with complications (n = 20). C1 hypertension—C10 diabetes cluster (n = 45) and C2 CVD—C7 PVD cluster (n = 30) were identified for female breast cancer patients. Gastric cancer patients also identified four comorbidity groups: C1 hypertension—C10 diabetes (n = 82), C2 CVD—C7 PVD (n = 61), C9 mild liver disease—C12 renal disease (n = 116), and C6 CHF—C8 COPD (n = 15). Three comorbidity clusters of rectal cancer patients were similar to those in patients with gastric cancer, which included: C1 hypertension—C10 diabetes cluster (n = 60), C2 CVD—C7 PVD cluster (n = 80), and C9 mild liver disease—C12 renal disease cluster (n = 81). C2 CVD—C7 PVD cluster (n = 57) and C9 mild liver disease—C12 renal disease cluster (n = 82) were for colon cancer patients.

### Step 4: examination of the associations between comorbidity patterns and outcome measures

Primary outcome measures of interest were treatment option and medical cost. Treatment options were divided into conventional treatment and targeted therapy according to the guidelines for the diagnosis and treatment of lung cancer, gastric cancer and breast cancer (2018 edition) [26] and colorectal cancer (2015 edition) [27] issued by the National Health Commission in China. Conventional treatment included surgical treatment, chemotherapy and radiotherapy. Surgical treatment for lung, gastric and colorectal cancer includes palliative and radical surgery, surgical treatment for invasive breast cancer includes modified radical surgery and breast-conserving surgery, and chemoradiotherapy includes (new) adjuvant chemoradiotherapy. Because targeted therapy is generally not the primary treatment plan for early and middle stage cancer patients, it was regarded as an optional treatment plan alone in this study. All treatment options were recoded as binary classification problems to determine whether patients received this type of therapy. Thirdly, the data of medical cost was directly extracted from the HIS system of the case hospital. The mean interpolation method was adopted, and the average total cost was 87,369.84 RMB.

Each cluster to patients without identified comorbidities was then compared using binary logistic regression with treatment options as the dependent variable, and linear regression models with medical cost as the dependent variable. All the models were adjusted for age, sex, education level, living area, and marriage status. IBM SPSS Statistics 26 was adopted for the cluster analysis and RStudio version 3.5.1 (Lucent Technologies, Murray Hill, NJ, USA) was used for the descriptive analysis and regression analysis in this study. Table 3 shows the multivariate-adjusted ORs and 95% CIs for treatment options and medical cost according to whether cancer patients with identified comorbidity cluster. For cancer treatment option, gastric cancer patients in the CHF—COPD (C6—C8) cluster chose less conventional treatment (OR = − 1.38; 95% CI − 2.46, − 0.17), lung cancer patients in the diabetes—diabetes with complications (C10—C13) cluster (OR = − 1.01; 95% CI − 1.89, − 0.30) and hypertension—CVD—PVD (C1—C2—C7) cluster (OR = − 0.87; 95% CI − 1.76, − 0.14) chose less targeted therapy. Rectal cancer patients in the hypertension—diabetes (C1—C10) cluster (OR = 17,347; 95% CI 665.8434028.15) had significantly higher medical cost during the treatment.

**Table 3:** Outcome measures of each comorbidity cluster compared against Chinese inpatient cancer patients with no comorbidity

	Conventional Treatment^#^	Targeted therapy^#^	Cost^##^
Without identified Cluster	1.00(reference)	1.00(reference)	\
**Lung Cancer**			
C6—C8 (N = 14)	0.47(− 0.80,2.09)	1.07(− 0.07,2.14)	32,385.1(31,623.33,97,589.41)
C1—C2—C7 (N = 74)	1.19(0.06,2.69)	− 0.87(− 1.76, − 0.14) *	− 1872.9(− 18,565.37,14,819.50)
C9—C12 (N = 56)	0.55(− 0.26,2.49)	− 0.14(− 0.66,0.33)	1629.9(− 11,376.49, 14,636.35)
C10—C13 (N = 20)	− 0.83(− 2.09,0.53)	− 1.01(− 1.89, − 0.30) *	− 3473.2 (− 18,648.53,11,702.18)
**Colon Cancer**			
C2—C7 (N = 57)	\	− 0.39(− 2.24,0.88)	14,161.1 (− 2776.94,31,099.08)
C9—C12 (N = 82)	\	− 0.14(− 1.41,0.88)	1575.9(− 13,186.62,16,338.41)
**Rectal Cancer**			
C1—C10 (N = 60)	\	− 0.44(− 3.36,1.25)	17,347.0 (665.84,34,028.15) *
C2—C7 (N = 80)	\	\	7944.0 (− 8795.22,24,683.14)
C9—C12 (N = 81)	\	− 1.09(− 4.00,0.58)	8437.6 (− 6192.26,23,067.53)
**Breast Cancer**			
C1—C10 (N = 45)	\	− 0.04(− 1.29,0.95)	− 5727.5 (− 21,918.75,10,463.74)
C2—C7 (N = 30)	\	0.33(− 0.93,1.34)	− 1754.6(− 21,015.74,17,506.54)
**Gastric Cancer**			
C1—C10 (N = 82)	0.02(− 0.64,0.78)	− 1.37(− 4.26,0.19)	− 7083.1(− 21,292.10,7125.96)
C2—C7 (N = 61)	0.56(− 0.30,1.63)	− 0.33(− 2.16,0.90)	− 4359.4 (− 20,549.02,11,830.23)
C9—C12 (N = 116)	− 0.11(− 0.66,0.51)	− 0.32(− 1.55,0.64)	6121.7(− 6079.63,18,322.93)
C6—C8 (N = 15)	− 1.38(− 2.46, − 0.17) *	0.49(− 2.43,2.16)	− 24,233.6(− 55,971.56,7504.42)

^**#**^ Binary logistic regression; ^**##**^ Linear regression; * *p* < 0.05; **p < 0.01; ***p < 0.001; all models were adjusted foe age, sex, education level, insurance status, marriage status and cancer stage; all colon, rectal and breast cancer patients received conventional treatment

## Possible applications

Firstly, the application of this framework could be highly beneficial at the local level. Identifying pattern of comorbidity can help oncologists detect and treat potential comorbid conditions at an early stage, preventing these conditions from worsening [11, 28]. This will facilitate the customization of more accurate treatment plans based on the specific cancer comorbidity patterns, enhancing treatment efficacy and reducing adverse reactions [29]. The integration of this framework with the multidisciplinary treatment (MDT) model will enhance the accuracy and effectiveness of cancer diagnosis and treatments. Guided by this framework, multidisciplinary teams can assess the disease from a systematic perspective, clarify the roles of various disciplines, and improve collaborative efficiency [30, 31]. Establishing uniform standards of inpatient cancer care based on identified comorbidity patterns, the overall survival rate and the quality of life for cancer patients could be improved [7]. Additionally, integrating this framework into HIS can improve resource utilization efficiency and prevent resource waste by allocating medical resources according to comorbidity patterns.

Secondly, the application of this framework could be impactful at regional and national levels. In terms of a national level, it can significantly reduce healthcare costs by addressing common comorbid conditions concurrently with cancer treatment. This integrated approach minimizes the need for multiple separate treatments and hospital visits, thereby reducing overall healthcare expenditure [32, 33]. National health databases can utilize comorbidity data for improving population health management, identifying at-risk groups and implementing early intervention programs. This preventative approach helps to reduce the incidence and severity of diseases across the population. In essence, identifying cancer comorbidity patterns can transform the landscape of healthcare at multiple levels. Hospitals can leverage this knowledge for better patient care, resource management, and team collaboration. At national level, this data supports robust public health initiatives, economic efficiencies, and scientific advancements [34]. Ultimately, this dual-level impact fosters a healthcare environment that is more responsive to the complexities of cancer and its associated conditions, leading to improved patient outcomes and a healthier society.

Thirdly, at the international level, the adaptability of this framework allows for its application in different countries. By understanding comorbidity patterns, international health organizations can develop standardized treatment protocols that address common health issues associated with cancer. This ensures that patients worldwide receive consistent, high-quality care regardless of where they are treated [35, 36]. Global health organizations, such as the World Health Organization (WHO), can use data on comorbidity patterns to design targeted intervention strategies that address both cancer and its associated conditions. These strategies can be tailored to specific regions based on prevalent health patterns, enhancing their effectiveness. These organizations can also advocate for policies that support integrated care for cancer patients with comorbidities. This includes promoting access to essential medicines, supporting health system strengthening, and ensuring that vulnerable populations receive the care they need. Identifying comorbidity patterns helps reduce healthcare costs by streamlining treatments and minimizing the need for multiple separate interventions [11, 37]. This contributes to more efficient use of global healthcare resources, which is particularly vital for low- and middle-income countries.

## Implementation challenges of the framework

**Fig. 5:**
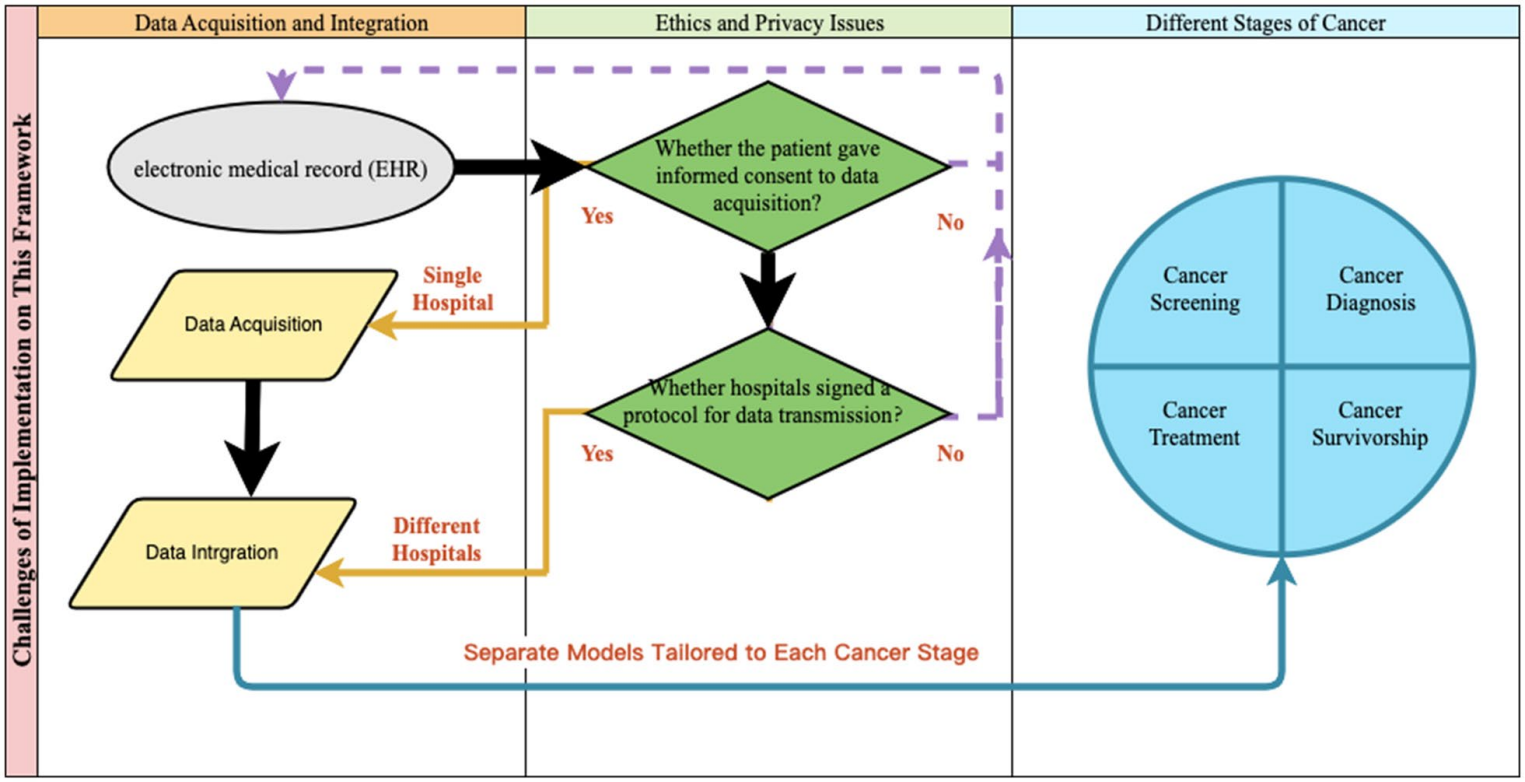
Flow chart of challenges of implementation on this framework

The challenges of implementation on this framework are illustrated in Fig. 5. The application of this framework needs to be optimized to overcome a few limitations in data acquisition and integration. At the local level, information in the electronic medical record (EHR) may contain inaccuracies or omissions at the time of entry which leads to incomplete records or data quality issues. Single hospitals may not have sufficient resources for continuously updated and maintained of the usage of the framework to ensure its accuracy and effectiveness. Between different hospitals, data recording systems and standards like ICD-10 might be different, leading to difficulties in data integration. Translation and standardization efforts may lead to misunderstandings or code errors due to inconsistent terminology. Differences in administrative structures and bureaucratic processes might slow down collaboration and implementation of the framework. Furthermore, hospitals varied widely across countries in IT infrastructure, data management systems and technical capabilities, which will affect the efficiency and quality of data collection, transmission and processing. The above disadvantages can be partially mitigated through the development of globally harmonized standards and protocols and enhanced international cooperation and communication. This will contribute to a more effective and practicable international application of the tumor comorbidity model framework.

The adoption of the framework might be constrained by ethics and privacy issues. The collection and processing of data involving detailed patient information might raise ethical and privacy protection challenges, particularly in the context of data sharing and multi-agency collaboration. Sharing patient data between hospitals raises significant ethical and legal challenges regarding patient consent and data privacy laws. Efficient and secure data communication between hospitals is critical but can be difficult to achieve, posing risks to data integrity and security. Ensuring secure and privacy-compliant data transmission across international borders requires stringent measures, which may be difficult to enforce consistently. To overcome these difficulties, several initiatives can be implemented. International organizations should work towards harmonizing ethical considerations and data protection laws globally to facilitate seamless data sharing while ensuring patient consent and privacy protection. Additionally, developed countries should support investments in healthcare technology and data infrastructure in countries with fewer resources to level the playing field for model implementation. Finally, stringent data security measures and international agreements should be implemented to ensure data integrity and protect patient privacy during cross-border data exchanges.

When applying the framework in patients at different stages of cancer, several disadvantages may arise. Patients at different cancer stages may exhibit varying levels of disease complexity, making it difficult to create a one-size-fits-all model. Tailoring the model to different cancer stages may require substantial resource allocation, including specialized staff, equipment, and time. Applying a uniform framework across different cancer stages without taking individual patient needs into account may raise ethical concerns regarding patient-specific care. Partitioning data according to cancer stages can lead to smaller sample sizes for each subgroup, potentially undermining the statistical power and reliability of the findings. The practical application of the framework may be limited if it does not integrate seamlessly with clinical workflows and decision-making processes, particularly as treatment priorities vary by stage. To overcome the disadvantages of applying the framework in patients at different stages of cancer, separate models tailored to each cancer stage could be developed to address the unique characteristics and treatment strategies associated with each stage, with rigorously testing and validating the models across different cancer stages to ensure accuracy, reliability, and clinical utility before full-scale deployment.

Some limitations should be acknowledged in this case study. One potential issue is the possibility of underestimating the prevalence and number of comorbidities. This stems from selecting cancer as the sole index disease for collecting comorbidity data, without considering scenarios where cancer might serve as a comorbidity and other diseases could be designated as the index disease. The tertiary hospital in China had the privilege of offering advanced therapeutic skills for diseases such as cancer within the hierarchical medical system, resulting in a lower proportion of cancer being included as an index disease for other conditions. Despite this limitation, the comorbidity rates in this study were consistent with other studies in Chinese inpatient cancer patients, so the findings in this study were relatively credible. Another limitation arises from the use of electronic medical records, which limits the ability to comprehensively include confounding factors such as body mass index, alcohol consumption, and smoking behaviors when exploring the association between comorbidity patterns and outcome measures. Based on this study, we will self-designed questionnaires by adding these factors in future studies to make a more detailed classification of different populations.

## Conclusions

This study developed a framework based on a case study to systematically explore the hidden information of comorbidity burden for inpatient cancer patients. Four steps constructed the framework: extraction of HIS data, identification of basic comorbidity characteristics, estimation of the comorbidity burden and examination of the associations between comorbidity patterns and outcome measures. Our study found that the most common comorbidities were hypertension, mild liver disease, renal diseases, diabetes and myocardial infarct among five main types of inpatient cancer patients in China. CVD—PVD cluster, mild liver disease—renal disease cluster and hypertension—diabetes cluster were the most prevalent. Cancer patients with cardiometabolic cluster had significantly higher medical cost and less treatment options than those without. This framework can be adopted to guide the patient care, hospital administration and medical resource allocation, and has the potential to be applied in various healthcare settings at local, regional, national, and international levels to foster a healthcare environment that is more responsive to the complexities of cancer and its associated conditions.

## Supplementary Information

Additional file 1.

Additional file 2.

## Data Availability

Data and materials are accessible upon the reasonable request to the research team.

## Abbreviations

HIS: Hospital information system
ICD-10: The international classification of diseases
CCI: Charlson comorbidity index
CVD: Cerebrovascular disease
PVD: Peripheral vascular disease
CHF: Congestive heart failure
COPD: Chronic obstructive pulmonary disease
EHR: Electronic medical record
