# Supplementary figures and images for: Developing a framework for estimating comorbidity burden of inpatient cancer patients based on a case study in China
Source: Glob Health Res Policy. 2025 Mar 3;10:13. doi: 10.1186/s41256-025-00411-3 (PMC11874831; doi:10.1186/s41256-025-00411-3)

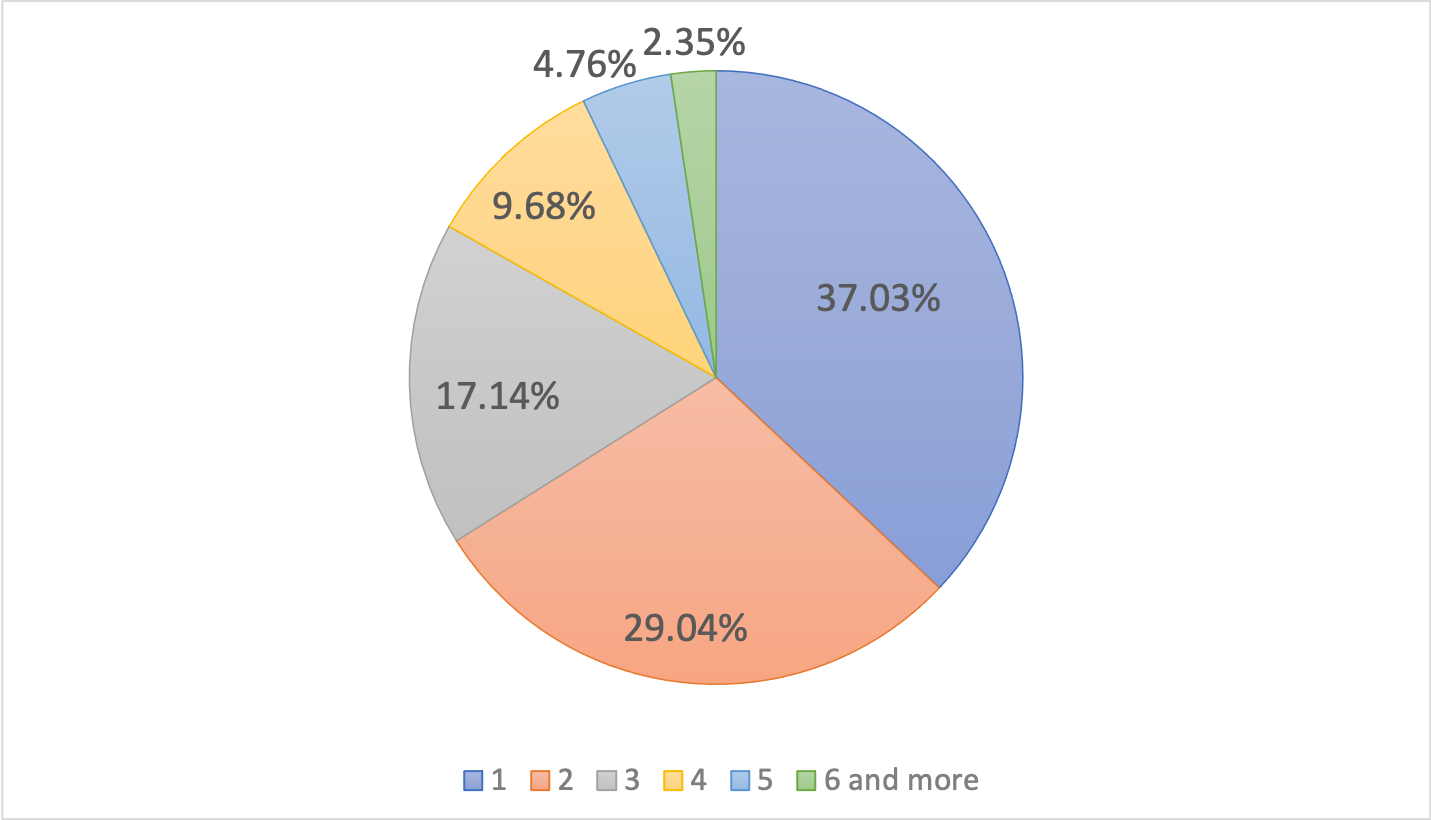

Supplement: Supplementary file 1 — Additional file 1. [file 41256_2025_411_MOESM1_ESM.png]
