# Supplementary material for: Developing a framework for estimating comorbidity burden of inpatient cancer patients based on a case study in China
Source: Glob Health Res Policy. 2025 Mar 3;10:13. doi: 10.1186/s41256-025-00411-3 (PMC11874831; doi:10.1186/s41256-025-00411-3)
Supplement: Supplementary file 2 — Additional file 2. [file 41256_2025_411_MOESM2_ESM.docx]

**Supplementary Tables**

| **Supplementary Table S1 Baseline characteristics of patients included in this study by Cancer Type** | | | | | | |
| --- | --- | --- | --- | --- | --- | --- |
|  | **Lung Cancer （N=1728）** | **Colon Cancer (N=570)** | **Rectal Cancer (N=582)** | **Breast Cancer (N=797)** | **Gastric Cancer (N=989)** | **P-value** |
| **Age, years, ﻿mean (SD)** | 63.33(10.36) | 64.87(12.16) | 65.34(10.60) | 54.40(12.06) | 64.16(10.82) | <0.001*** |
| **Cost, RMB, ﻿mean (SD)** | 72641.68(71505.00) | 80289.67(61457.284) | 86830.67(62634.65) | 51722.40(52114.15) | 81035.65(62819.63) | <0.001*** |
| **Sex, n (%)** |  |  |  |  |  | <0.001*** |
| Female | 790 (45.72) | 245 (42.98) | 226 (38.83) | 797 (100.00) | 260 (26.29) |  |
| Male | 938 (54.28) | 325 (57.02) | 356(61.17) | 0 (0.00) | 729 (73.71) |  |
| **Insured, n (%)** |  |  |  |  |  | 0.22 |
| Yes | 1641 (94.97) | 541 (94.91) | 544 (93.47) | 746 (93.60) | 946 (95.65) |  |
| No | 87 (5.03) | 29 (5.09) | 38(6.53) | 51 (6.40) | 43 (4.35) |  |
| **Married, n (%)** |  |  |  |  |  | <0.001*** |
| Yes | 1528(88.43) | 509 (89.30) | 496 (85.22) | 724 (90.84) | 837 (84.63) |  |
| No | 200 (11.57) | 61 (10.70) | 86(14.78) | 73(9.16) | 152 (15.37) |  |
| **﻿Residency, n (%)** |  |  |  |  |  | <0.001*** |
| Urban | 1107(64.06) | 433 (75.96) | 363(62.37) | 497 (62.36) | 631 (63.80) |  |
| Rural | 621 (35.94) | 137(24.04) | 219(37.63) | 300(37.64) | 358(36.20) |  |
| **Cancer stage, n (%)** |  |  |  |  |  | <0.001*** |
| 0 | 95（5.50） | 0（0.00） | 0（0.00） | 29（3.64） | 0（0.00） |  |
| Ⅰ | 624（36.11） | 93（16.32） | 135（23.20） | 278（34.88） | 191（19.31） |  |
| Ⅱ | 151（8.74） | 201（35.26） | 142（24.40） | 316（39.65） | 187（18.91） |  |
| Ⅲ | 328（18.98） | 211（37.02） | 261（44.85） | 163（20.45） | 395（39.94） |  |
| Ⅳ | 530（30.67） | 65（11.40） | 44（7.56） | 11（1.38） | 216（21.84） |  |
| **Conventional treatment, n (%)** |  |  |  |  |  | <0.001*** |
| Yes | 1503(86.98) | 570(100.00) | 582(100.00) | 797(100.00) | 863(87.26) |  |
| No | 225(13.02) | 0(0.00) | 0(0.00) | 0(0.00) | 126(12.74) |  |
| **Targeted therapy, n (%)** |  |  |  |  |  | <0.001*** |
| Yes | 295(17.07) | 28(4.91) | 17() | 100(12.55) | 41(4.15) |  |
| No | 1433(82.93) | 542(95.09) | 565() | 697(87.45) | 948(95.85) |  |
| *** P-value < 0.001; ** P-value <0.01; * P-value <0.05  Numbers presented are n (%) unless otherwise specified | |  |  |  |  |  |

| **Supplementary Table S2 Baseline characteristics of cancer patients included in this study by comorbidity number and severity** | | | | | | | |
| --- | --- | --- | --- | --- | --- | --- | --- |
|  | **Number of Comorbidity** | | | **Severity of Comorbidity** | | | |
|  | **<3   (N=3460)** | **﻿≥3  (N=1206)** | **P-value** | **Mild  (N=2456)** | **Moderate (N=1358)** | **Severe (N=852)** | **P-value** |
| **Age, years, ﻿mean (SD)** | 60.43(11.64) | 68.13(9.49) | <0.001*** | 59.40(11.93) | 66.45(9.94) | 64.68(10.72) | <0.001*** |
| **Cost, RMB, ﻿mean (SD)** | 71088.71(63008.45) | 80618.13(70978.73) | <0.001*** | 67767.54(55960.04) | 77574.72(58391.37) | 83813.23(93098.56) | <0.001*** |
| **Sex, n (%)** |  |  | <0.001*** | 34211.52(7173.71) |  |  | <0.001*** |
| Female | 1839 (53.15) | 479 (39.72) |  | 1458 (59.36) | 550 (40.50) | 310 (36.38) |  |
| Male | 1621 (46.85) | 727 (60.28) |  | 998 (40.64) | 808(59.50) | 542 (63.62) |  |
| **Insured, n (%)** |  |  | <0.01** |  |  |  | <0.001*** |
| Yes | 3256 (94.10) | 1162 (96.35) |  | 2299 (93.61) | 1305 (96.10) | 814 (95.54) |  |
| No | 204 (5.90) | 44 (3.65) |  | 157 (6.39) | 53 (3.90) | 38 (4.46) |  |
| **Married, n (%)** |  |  | <0.001*** |  |  |  | <0.001*** |
| Yes | 3000 (86.71) | 1094(90.71) |  | 2130 (86.73) | 1215(89.47) | 749(87.91) |  |
| No | 460 (13.29) | 112(9.29) |  | 326 (13.27) | 143(10.53) | 103(12.09) |  |
| **﻿Residency, n (%)** |  |  | <0.001*** |  |  |  | <0.001*** |
| Urban | 2083 (60.20) | 948 (78.61) |  | 1467 (59.73) | 989 (72.83) | 575 (67.49) |  |
| Rural | 1377(39.80) | 258 (21.39) |  | 989(40.27) | 369 (27.17) | 277 (32.51) |  |
| **﻿Cancer type, n (%)** |  |  | <0.001*** |  |  |  | <0.001*** |
| Lung Cancer | 1196 (34.57) | 532 (44.11) |  | 745 (30.33) | 500 (36.82) | 483 (56.69) |  |
| Colon cancer | 370 (10.69) | 200 (16.58) |  | 255 (10.38) | 240 (17.67) | 75 (8.8) |  |
| Rectal cancer | 421 (12.17) | 161 (13.35) |  | 303 (12.34) | 223 (16.42) | 56 (6.57) |  |
| Breast Cancer | 738 (21.33) | 59 (4.89) |  | 675 (21.48) | 99 (7.29) | 23 (2.70) |  |
| Gastric Cancer | 735 (21.24) | 254 (21.06) |  | 478 (19.46) | 296 (21.80) | 215(25.23) |  |
| **Cancer stage, n (%)** |  |  | <0.001*** |  |  |  | <0.001*** |
| 0 | 91 (2.63) | 33 (2.74) |  | 78 (3.18) | 42(3.09) | 4(0.47) |  |
| Ⅰ | 1003 (28.99) | 318 (26.37) |  | 849 (34.57) | 433 (31.89) | 39 (4.58) |  |
| Ⅱ | 793 (22.92) | 204 (16.92) |  | 647 (26.34) | 306 (22.53) | 44 (5.16) |  |
| Ⅲ | 1017 (29.39) | 341 (28.28) |  | 793 (32.29) | 495 (36.45) | 70 (8.21) |  |
| Ⅳ | 556 16.07) | 310 (25.70) |  | 89(3.62) | 82 (6.04) | 695 (81.57) |  |
| **Conventional treatment, n (%)** |  |  | 0.001** |  |  |  | <0.001*** |
| Yes | 3227(93.27) | 1088(90.22) |  | 2352(95.77) | 1279(94.18) | 684(80.28) |  |
| No | 233(6.73) | 118(9.78) |  | 104(4.23) | 79(5.82) | 168(19.72) |  |
| **Targeted therapy, n (%)** |  |  | 0.34 |  |  |  | <0.001*** |
| Yes | 348(10.06) | 133(11.03) |  | 143(5.82) | 55(4.05) | 283(33.22) |  |
| No | 3112(89.94) | 1073(88.97) |  | 2313(94.18) | 1303(95.95) | 569(66.78) |  |
| *** P-value < 0.001; ** P-value <0.01; * P-value <0.05 | | | | | | | |
| Numbers presented are n (%) unless otherwise specified | | | | | | | |
